# Antimicrobial resistance in the United States: Origins and future directions

**DOI:** 10.1017/S0950268824000244

**Published:** 2024-02-12

**Authors:** Kent F. Sutton, Lucas W. Ashley

**Affiliations:** 1 Duke University School of Medicine, Duke University Medical Center, Durham, NC, USA; 2 Brody School of Medicine at East Carolina University, Greenville, NC, USA

**Keywords:** antimicrobial resistance, bacterial infections, health policy, infectious disease control, public health

## Abstract

Antimicrobial resistance (AMR) remains a critical public health problem that pervades hospitals and health systems worldwide. The ongoing AMR crisis is not only concerning for patient care but also healthcare delivery and quality. This article outlines key components of the origins of AMR in the United States and how it presents across the American healthcare system. Numerous factors contributed to the crisis, including agricultural antibiotic use, wasteful prescribing practices in health care, conflicting behaviours among patients and clinicians, patient demand and satisfaction, and payment and reimbursement models that incentivize inappropriate antibiotic use. To combat AMR, clinicians, healthcare professionals, and legislators must continue to promote and implement innovative solutions, including antibiotic stewardship programmes (ASPs), hand hygiene protocols, ample supply of personal protective equipment (PPE), standardized treatment guidelines for antibiotic prescribing, clinician and patient educational programmes, and health policy initiatives. With the rising prevalence of multi-drug resistant bacterial infections, AMR must become a greater priority to policymakers and healthcare stakeholders.

## Introduction

Antimicrobial resistance (AMR) is an ongoing urgent threat to the American health system with considerable public health relevance. Resistance to life-saving antibiotics warrants a thorough investigation of the variables fuelling the crisis and an analysis of the feasibility of current solutions. Antibiotic over-prescription and overuse drive AMR, as ‘overuse’ antibiotics may refer to those prescribed unnecessarily, with undue duration, and for clinical indications unsupported by evidence [1, 2]. A study of 21,825 patients conducted from 2017–2019 found nearly half of them received overuse prescriptions [1]. In 2009 alone, antibiotic-related expenditures exceeded $10 billion [3]. $10 billion is a relatively minor amount compared to the $2.6 trillion spent on health care in the U.S.A in 2009 [4], but recent estimates suggest at least 30% of antibiotics prescribed are inappropriate [5], demonstrating the high degree to which antibiotic overuse contributes to medical waste and inefficiency in health care. Antibiotic overuse is largely to blame for multi-drug resistant bacterial infections, such as methicillin-resistant *Staphylococcus aureus* (MRSA), vancomycin-resistant enterococci (VRE), *Streptococcus pneumoniae*, and numerous gram-negative bacteria [2, 6–10]. These pathogens infect 2.8 million people and kill an estimated 35,000 Americans annually, costing an estimated $35 billion [2, 11, 12].

Multi-drug resistant bacterial infections pose consequences for the health system in the form of high medical costs, worsening mortality outcomes, food insecurity, shrinking availability of efficacious drugs, in addition to consequences for individual patients, including long and costly hospitalizations and increased risk for recurrent infections [13]. Clinicians now struggle to care for patients with conditions such as pneumonia, bacteraemia, sexually transmitted infections, and foodborne illnesses [13]. Addressing AMR must be a priority to the U.S. health system, and government officials, policymakers, and clinicians must ensure patients receive appropriate and necessary care while also working to prevent and contain future epidemics. This work aims to characterize the antimicrobial resistance crisis in health care, discuss causes of the problem and how they are presented in the health system, and analyse policy implications that may improve antibiotic stewardship. The current literature suggests the AMR crisis arose from widespread agricultural antibiotic use, wasteful prescribing practices in health care, systemic, maladaptive behaviours among patients and clinicians, ill-informed patient demand, and inefficient payment and reimbursement models that incentivize improper antibiotic overuse.

## Agriculture

Antibiotic overuse and AMR are complex issues with an array of contributing variables in the health system. The ubiquitous nontherapeutic use of antibiotics in agriculture played a pivotal role in America’s AMR problem by creating a ‘reservoir of resistance’ [14]. The use of antibiotics in agriculture for growth promotion and infection control and prevention among livestock dates to the 1940s [15]. Antimicrobial preservatives and other additives in food products are also partly to blame for the evolving trends in AMR [16]. These practices have implications ‘at every stage of the food chain, from farm to fork’ [17]. Agricultural use of antibiotics fuels AMR via direct mechanisms, such as human consumption of animal food products, and indirect mechanisms, such as agricultural and animal waste runoff into surrounding environments [18]. The contribution of agricultural antibiotic misuse to environmental antimicrobial resistance was deemed so consequential that it culminated in the development of the term ‘antibiotic resistome’, which refers to all the antibiotic resistance genes [19]. Unnecessary agriculture exploitation of antibiotics led to the establishment of the Global Action Plan on Antimicrobial Resistance, a partnership of the WHO, World Organization for Animal Health, and Food and Agriculture Organization of the United Nations. This effort focused on strengthening surveillance of antimicrobial consumption among humans and animals and improving research strategies to delineate downstream effects on food supply and the environment [20].

## Clinician and patient behaviour

While food suppliers relied on antibiotics in cultivating crops and livestock on farms, physicians and patients in clinics and hospitals, along with evolving ‘cultural expectations’ in health care [21], also helped to fuel AMR through antibiotic over-prescription [22]. Adoption of a business-minded approach in healthcare delivery represents a ‘burgeoning era of “pay for performance”’ [23, 24]. A pay for performance model prioritizes a particular approach to ‘patient satisfaction’ and transforms health care into a transaction between clinicians and patients. ‘Patient satisfaction’ refers to a phenomenon that is intimately connected to patient demand: patients increasingly want a ‘tangible product from a clinical encounter’ [25]. Primary care providers frequently identify patient demand as a leading determinant of antibiotic prescribing [26]. Providers who do not authorize antibiotic treatment may find themselves with unsatisfied patients, even though patients regularly present with misinformed notions about antibiotics and are often unaware of these drugs’ inability to treat viral infections [25]. In these cases, antibiotic treatment would be a form of low-value care and could cause potential harm. Nonetheless, poor patient satisfaction may damage the patient–provider relationship and weaken a practice’s ability to maintain its patient population. Clinicians have a self-interest in developing rapport with patients by satisfying wishes for antibiotic prescriptions even when they are unnecessary [25]. In this sense, clinicians may maintain their practice through antibiotic over-prescription to ‘avoid being perceived as doing nothing’ [27]. Clinicians also perpetuate antibiotic over-prescription by acting on a belief that ‘patients who really want antibiotics will obtain them anyway’ [27, 28]. This is a reasonable assumption with the rise in popularity of urgent care centres and telemedicine services, which provide patients numerous avenues to obtain a prescription. Indeed, where a patient chooses to seek care influences the provision of antibiotics; DePew et al. found that patients at community health centres and emergency departments were more likely to receive antibiotics [29]. Determined patients exhibiting these behaviours and providers willing to write prescriptions are systemic features across the American health system and underscore how behaviour contributes to antibiotic over-prescription.

## Health system administration

Clinician and patient behaviour are partly to blame for the growing AMR crisis, but their habits are likely informed by top-down institutional policies and procedures. Institutional comfort level with diagnostic uncertainty may either encourage or discourage initiation of empiric antibiotics through official or unspoken hospital guidelines or health system policies [1]. This phenomenon is particularly relevant to nursing homes that care for elderly populations, where providers regularly treat asymptomatic bacteriuria with antibiotics, despite no evidence supporting treatment [30]. In this regard, providers initiate antibiotic treatment for bacteriuria on questionable grounds, rooted in caution and common practice, rather than evidence-based medicine. Even when evidence-based indications for antibiotics exist, organizational procedures frequently fail to track antibiotic use after delivery of services, especially during transitions of care such as hospital discharge [1]. Community pharmacists lack validated instruments to monitor patient adherence to proper treatment regimens, which weakens our ability to evaluate antibiotic effectiveness and further fuels AMR [31].

The interplay between institutional policies made at the top and individual behaviour at the clinician level also reflects the reality that clinicians may disagree with institutional recommendations on antibiotic selection and discontinuation [6]. Positive associations between number of years since a clinician’s training and antibiotic over-prescription may help to explain why such disagreements about best prescribing practices arise [32]. Clinicians farther from their training may not be abreast of recent trends in AMR and literature detailing the harms of over-prescription and promoting the need for antibiotic stewardship [6]. Clinicians may resist antibiotic stewardship task forces due to feelings of resentment over loss of autonomy in clinical management [6]. Resentment and resistance to stewardship activities among clinicians may contribute to over-prescription and hinder collective action against AMR.

## Payment & reimbursement models

Although patient and clinician behaviour, systemic expectations in health care, institutional practices, and agricultural antimicrobial overuse all contribute to AMR, this analysis would be incomplete without a thoughtful discussion of how healthcare payment models and insurance coverage contributed to the problem. An analysis of the 2012 National Ambulatory Medical Care Survey found that patients with health insurance were more likely to receive antibiotic prescriptions [29]. This finding reflects the reality that insurance status, ability to pay, and fee-for-service models in the American healthcare system are partly to blame for over-prescription and AMR. Numerous scholars have studied how policies embedded in Medicare may have been complicit in the rise of AMR. Medicare’s reimbursement model for inpatient treatment and services relies on inpatient payment bundles, also known as diagnosis-related groups (DRGs), which consist of all services clinicians provide for each diagnosis, including antibiotic prescriptions. Hospitals receive a fixed payment for these collective services, which makes older, less effective generic antibiotics more economical instead of newer, more effective, albeit expensive, drugs [33–35]. Innovative drugs may not be cost-effective for hospitals, given low reimbursement through Medicare’s DRG system, and clinicians are unlikely to write such prescriptions when faced with the possibility of angering hospital leadership and impacting their institution’s bottom line [36]. In this way, Medicare policies encourage cost-saving mechanisms that further embolden improper antibiotic prescribing patterns in inpatient settings and may prevent patients from receiving the best possible treatment.

Second, Medicare’s volume-based model for reimbursement has concerning implications for drug development for multi-drug resistant infections. The patient population requiring innovative antibiotics for multi-drug resistant infections is fairly small, which may translate to lower revenue potential for pharmaceutical companies that must invest enormous time, money, and human capital to develop experimental drugs [33, 37, 38]. Both Medicare’s payment model and antibiotic stewardship itself, grounded by the goal of curtailing antibiotic consumption overall, shrink volume and revenue projections [33, 35, 36]. In a utopic society, where the good of the patient takes precedence, integrity and morality alone may inspire corporations to develop drugs that patients need most. However, poor revenue potential is a non-starter for corporations, and disincentives ingrained in Medicare may have discouraged antibiotic R&D and contributed to our current struggle to combat the deadliest infections and address AMR [33, 36, 38]. Several corporations, including Novartis, discontinued their antibiotic drug development programmes altogether, while others declared bankruptcy [36, 39]. These policies and practices have been especially concerning for Medicare beneficiaries, such as elderly and vulnerable patients with disabilities and chronic conditions, who are disproportionally affected by AMR infections and deaths [33, 40]. Without effective medications to treat patients with multi-drug resistant infections, the U.S.A will continue to report skyrocketing healthcare utilization costs and hospitalization rates [33, 41, 42]. Medicare is not innocent in the AMR crisis, and unfortunately, the people it strives to protect likely bear the brunt of the problem.

## Solutions and policy implications

This analysis identified long-standing agricultural practices, systemic patient and clinician behaviours, patient demand and satisfaction, organizational policies and hierarchies, fee-for-service payment models, and two specific Medicare policies as critical variables that contributed to antibiotic over-prescription and antimicrobial resistance in the U.S.A. In 2014, the CDC announced a national effort to tackle AMR by announcing the Core Elements of Hospital Antibiotic Stewardship Programs to promote AMR awareness and inspire thoughtful change in prescribing patterns [43]. In 2019, the Centers for Medicare and Medicaid Services (CMS) established requirements for hospital antibiotic stewardship programmes in facilities seeking reimbursement for Medicare and Medicaid nationwide [43]. The Joint Commission mandated an antibiotic stewardship requirement in outpatient settings in 2020 [44]. Barlam et al. estimated 89% of hospitals implemented all seven CDC Core Elements by 2019 [45]. However, the feasibility of solutions proposed in these initiatives requires further analysis.

Antibiotic stewardship priorities include improvement of diagnostic tools, evidence-based standardization of antibiotic selection and treatment duration, enhanced data monitoring at care transitions such as hospital discharge, and creation of annual antibiograms [2, 45, 46]. Equally important to stewardship and infection prevention are basic cleanliness practices, such as hand washing, proper donning of PPE, and disinfection of healthcare tools and devices [13]. A growing literature emphasizes the success of ASPs, including results demonstrating ASPs’ role in reducing length of hospital stays and adverse events from antibiotics, in addition to associations between ASPs and lower *C. difficile* infection rates [13, 47]. The development of standardized treatment guidelines for common conditions that may require antibiotics is another popular element of ASPs. Standardized treatment protocols embedded in electronic medical records have been successful in enforcing proper antibiotic dosing and reducing incorrect prescription orders [48]. In this way, greater reliance on standardized treatment protocols in ASPs may help to address the AMR crisis and improve patient safety while also making the provision of antibiotics more efficient, cost-effective, and evidence-based.

Educational materials and programming for both patients and clinicians are commonly included in ASPs, as such interventions demonstrated success in reducing antibiotic prescribing [6, 27]. Experts recommend clinicians educate patients about the expected course of illnesses, explain why antibiotics will not be effective, discuss potential antibiotic side effects and drug interactions, and impart anticipatory guidance for when patients should return to clinic for further evaluation [27]. Educational initiatives may bridge patients’ knowledge gaps in proper antibiotic use and engender more mutual understanding between patients and clinicians. Although educational initiatives may be another responsibility for busy providers, clinician-mediated education could be seamlessly implemented into infection-related clinic visits. This approach has the potential to promote shared decision-making between patients and providers, save time in future clinic visits, and reduce the burden of unnecessary appointments for common colds. In addition, outpatient clinician-mediated education may benefit from wider adoption of delayed prescription, which affords patients the opportunity to receive a prescription days after their appointment if they are not satisfied with their symptomatic progress [49]. This practice enables clinicians to make a tangible promise without the pressure of prescribing antibiotics outright and to provide time for patients’ symptoms to improve [49]. Delayed prescription is promising, as it is a reasonable compromise between clinicians and patients and could reduce over-prescription [50].

Public policy could be another powerful vehicle to combat AMR. The U.S.A enacted a handful of laws with tangible solutions that target AMR over the past decade. The 2012 FDA Safety and Innovation Act established the Generating Antibiotic Incentives Now (GAIN) initiative, which supported research and development of new drugs, approved more rapid drug review protocols, and strengthened antibiotic stewardship activities at the national level [51]. The 21^st^ Century Cures Act of 2016 built on this 2012 law by authorizing a new approval process for drugs designed specifically for a ‘limited population’, which includes innovative medications that may treat drug-resistant infections without existing therapeutic options [52]. Additionally, the Pandemic and All Hazards Preparedness and Advancing Innovation Act of 2019 addressed AMR from a biodefense perspective and codified the Presidential Advisory Council on Combating Antibiotic-Resistant Bacteria (PACCARB) [53, 54]. These successful pieces of legislation were critical milestones in the fight against AMR, and the U.S.A would be prudent to exercise public policy to further combat agricultural antimicrobial consumption in particular. The U.S. Congress should revisit two bills, the Preventing Antibiotic Resistance Act of 2013 introduced by Senator Diane Feinstein, and the Preservation of Antibiotics for Medical Treatment Act of 2017 introduced by the late Congresswoman Louise McIntosh, both of which strive to safeguard antimicrobial devices for the treatment of ill Americans and reduce unnecessary antimicrobial use in agriculture [55, 56].

## Conclusion

America’s public policy efforts have been bolstered by recent efforts at CMS, the CDC, and the Joint Commission. Medicare should continue to exercise surveillance of antibiotic stewardship through punitive measures, as the ACA mandates financial penalties for hospitals with high infection rates related to procedures, indwelling catheters, and central line tubes [57]. Additionally, as CMS is the largest healthcare payer in the United States, modifications to payment practices in Medicare are essential to addressing antibiotic stewardship and AMR. As of 2019, CMS now permits hospitals to code AMR under a higher severity designation, which improves reimbursement for treatment of multi-drug resistant infections and allows clinicians to prescribe more effective and innovative antibiotics that otherwise would not be cost-effective [33, 58]. Although this is a positive step, scholars and healthcare professionals called on CMS to address AMR even further by withdrawing antibiotics from DRG payment plans and improve reimbursement for inpatient antibiotic use [36]. In 2020, CMS responded by expanding reimbursement for new antibiotics proven to be more effective than currently offered drugs [59]. These changes by CMS, coupled with the FDA’s 2021 updated policy enabling faster review of experimental antibiotics [60], may represent a new frontier in the fight against AMR, specifically by expanding drug options, incentivizing enhanced treatment of multi-drug resistant infections, and hopefully preventing future epidemics or at a minimum preparing the U.S.A to contain epidemics when they arise. These solutions, in conjunction with strengthened antibiotic stewardship activities, have the potential to aid the U.S. health system in fighting big bugs with the right drugs – no more and no less.
